# Potential Roles of Long Noncoding RNAs as Therapeutic Targets in Organ Transplantation

**DOI:** 10.3389/fimmu.2022.835746

**Published:** 2022-03-11

**Authors:** Saeedeh Salehi, Shima Afzali, Abbas Shahi, Ali Akbar Amirzargar, Yaser Mansoori

**Affiliations:** ^1^ Department of Immunology, School of Medicine, Tehran University of Medical Sciences, Tehran, Iran; ^2^ Noncommunicable Diseases Research Center, Fasa University of Medical Sciences, Fasa, Iran; ^3^ Department of Medical Genetics, Fasa University of Medical Sciences, Fasa, Iran

**Keywords:** long noncoding RNA, transplantation, graft rejection, biomarker, solid organ

## Abstract

Organ transplantation is the most preferred treatment option for end-stage organ diseases; however, allograft rejection is the major hurdle in successful long-term transplant survival. In spite of developing better HLA matching and more effective immunosuppressive regimen, one-year graft survival has been increased by nearly 90% and the incidence of acute rejection by one-year post-transplantation has been decreased by 12.2% in the last decades, chronic allograft rejection has remained as one of the major obstacles to the long-lasting survival of the transplanted allograft. Therefore, seemingly preventing the allograft rejection and inducing immunological tolerance against transplanted allografts is one of the primary goals in transplantation research to enable long-lasting graft survival. Various mechanisms such as long noncoding RNAs (lncRNAs) have been proposed that induce immune tolerance by modulating the gene expression and regulating innate and adaptive immune responses during transplantation. Besides, because of involvement in regulating epigenetic, transcriptional, and post-translational mechanisms, lncRNAs could affect allograft status. Therefore, these molecules could be considered as the potential targets for prediction, prognosis, diagnosis, and treatment of graft rejection. It is suggested that the noninvasive predictive biomarkers hold promise to overcome the current limitations of conventional tissue biopsy in the diagnosis of rejection. Hence, this review aims to provide a comprehensive overview of lncRNAs and their function to facilitate diagnosis, prognosis, and prediction of the risk of graft rejection, and the suggestive therapeutic choices after transplantation.

## 1 Introduction

### 1.1 Transplantation Perspective

Solid-organ transplantation is the most preferred treatment option for end-stage organ failure and could ameliorate life expectancy and quality, but allograft rejection is still the main barrier to long-term graft survival. Although widespread use of conventional immunosuppressive drug regimens and improvement in surgical techniques, HLA matching and cross-matching technologies have significantly decreased the rate of allograft rejection, induction of the immunological tolerance and the long-lasting survival of transplanted organs have remained as the main challenges. Based on the 2019 annual data report of organ procurement and transplantation network (OPTN), the incidence of acute rejection by one year post-transplantation were 7% (1), 16% (2), 25.1% (3), 25.4% (4), 37.4% (5), and 12% (6) for Kidney, lung, heart, liver, intestine, and all categories of pancreas transplantation, respectively. Currently, routine monitoring of transplant recipients includes the measurement of biochemical parameters such as serum creatinine and protein excretion for renal transplant recipients, bilirubin and liver enzymes level consisting of alanine aminotransferase (ALT), aspartate aminotransferase (AST), gamma-glutamyl transferase (GGT) for hepatic transplantation, and N-terminal pro-hormone of brain natriuretic peptide (NT-proBNP), and cardiac troponin for heart transplant recipients (7, 8). But these markers are insensitive and non-specific and only detectable when the allograft dysfunction has occurred. Furthermore, accurate diagnosis of acute rejection requires allograft biopsy to confirm the underlying pathologies. Although the tissue biopsy is expensive, painful, and risky procedure and is limited by sampling error, inter-observer discrepancy in grading, and complications such as bleeding, infection, and allograft damage, currently it has been considered as a gold standard technique for investigating the graft status (9). Therefore, it is needed to identify a reliable and noninvasive methods that could replace biopsy or decrease the dependency on biopsy reporting. Hopefully, biomarkers in biofluids can be used to monitor the recipient’s immune response to the allograft in the near future. Noncoding RNAs (ncRNAs) are attracting widespread interest due to their ability in affecting the biological pathways which play important roles in health and disease. Besides, their stability in tissue and biofluids potentiates them as the appropriate candidates for biomarker discovery.

A growing body of evidence suggests that long non-coding RNAs (lncRNAs) have been participated in developmental, biological, and pathological processes through affecting various molecular pathways, such as transcription, post-transcription, and translation levels. In the rejection processes, the pathologic process could be detected at molecular level before occurrence of histological or clinical manifestations. Several investigations were carried out for profiling of lncRNAs in serum, plasma and urine samples of transplant rejected patients to find potential biomarkers for prognosis, diagnosis, and treatment of graft rejection (10–16).

To the best of our knowledge, no secondary study has assessed the role of lncRNAs in organ transplantation. Therefore, we aimed to conduct a comprehensive literature search and to summarize important results that coming from the original research which have evaluated the lncRNAs and their performance as predictive and/or diagnostic biomarkers for organ transplantation.

### 1.2 Noncoding RNAs

**Figure 1:**
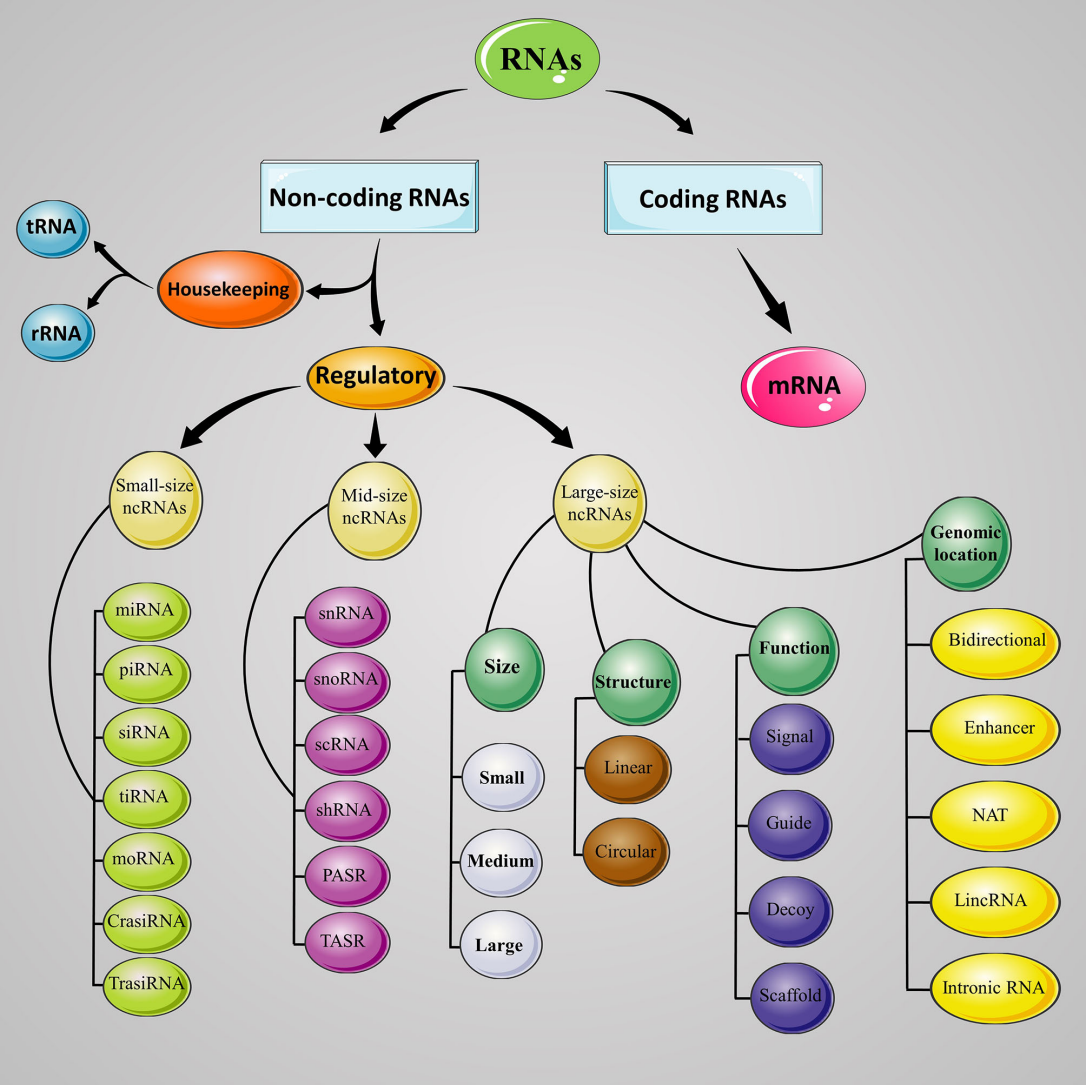
Classification of ncRNAs. Schematic demonstrates the lncRNAs classes and sub-classes based on their size, genomic location, function, and structure. RNA, ribonucleic acid; ncRNA, noncoding RNA; mRNA, messenger RNA; tRNA, transfer RNA; rRNA, ribosomal RNA; miRNA, microRNA; piRNA, Piwi-interacting RNA; siRNA, small interfering RNA; tiRNA, tRNA-derived stress-induced RNA; moRNA, microRNA offset RNA; crasiRNA, centromere repeat-associated short interacting RNA; snRNA, small nuclear RNA; snoRNA, small nucleolar RNA; scRNA, small conditional RNA; PASR, promoter-associated small *RNA; TASR*, terminator-associated small *RNA; NAT, n*atural antisense transcript; lincRNA, long intergenic noncoding RNA.

ENCODE (Encyclopedia of DNA Element) project has demonstrated that about 80% of the human genome is actively transcribed to at least one RNA, while only a small fraction (less than 3%) of them translate to the protein, and the remaining are non-protein-coding genes which transcribed but do not translate to the protein, consist of non-coding RNAs (ncRNAs) (17). ncRNA’s functions generally include regulation of gene expression at the transcriptional and post-transcriptional level. The ncRNAs are categorized into housekeeping and regulatory RNAs. Transfer RNAs (tRNAs) and ribosomal RNAs (rRNAs) are two important examples of housekeeping ncRNAs which involved in protein synthesis. ncRNAs with regulatory function are mainly divided into three groups; (I) Small non-coding RNAs (sncRNA; up to 50 nucleotides) such as small interfering RNA (siRNA), microRNA (miRNA) and piwi-interacting RNA (piRNA), (II) mid-size non-coding RNAs (mncRNAs; 50 to 400 nucleotides) include small nuclear RNAs (snRNAs), small nucleolar RNAs (snoRNA), small conditional RNA (scRNA), and short hairpin RNA (shRNA), and (III) long non-coding RNAs (lncRNAs; more than 400 nucleotides) (18–20). Schematic representation of ncRNAs has been depicted in **Figure 1**.

#### 1.2.1 History, Characteristics and Classification of lncRNAs

It has been revealed that the human genome is highly dynamic, and just 2.2% of the DNA sequence is conserved. Interestingly, more than 80% of lncRNAs are among the least conserved genes. The lncRNA H19 is the first non-coding gene discovered in 1989; however, it was classified as mRNA at that time.

There are various mechanisms that lncRNAs seemed to arise from, including alterations in protein-coding genes, such as losing the protein-coding potential, retrotransposition of a noncoding gene, chromosomal rearrangement, duplicating the already existing lncRNA sequences, as well as the transposable element which can make lncRNA by polyadenylation, splicing, RNA editing. Although the *de novo* origin of lncRNAs is difficult to prove; there are a few examples for it, such as Polidi that is a testis-specific lncRNA (21, 22).

LncRNAs are described as non-coding transcripts that are at least 200 nucleotides in length and could be find in nucleus, cytoplasm, and other compartments. These molecules are functional RNA elements that are normally expressed at low levels in a tissue-specific and time-limited mode, with half-lives varying from < 2h to > 16h and a median half-life of 3.5h. Although these molecules are poorly conserved among various species, their particular secondary structures, localization, and mechanisms of action appear to be highly conserved (23–25).

LncRNAs can be classified into five groups based on their location and proximity to protein-coding sequence, comprising of long intergenic noncoding RNAs (lincRNAs), sense or exonic, intronic, natural antisense transcript (NATs), and bidirectional or divergent lncRNAs (26, 27), or classified to four archetypes based on mechanism of action, including signal, decoy, guide, and scaffold (23, 28), or categorized to three groups based on their length, small lncRNA with 200 to 950 nucleotides, medium lncRNA with 950 to 4800 nucleotide, and large lncRNA with more than 4800 nucleotides (**Figure 1**). Small (58%) and medium (78%) lncRNAs are major lncRNA in the human and mouse, respectively (29). In humans, there are from 17948 lncRNAs predicted by GENCODE (30) to 96411 predicted by NONCODE (31). These differences between these two databases highlight either the massive number of lncRNA genes in the human genome and the growing need for ongoing lncRNAs research to discover these inter-databases discrepancies.

#### 1.2.2 Biogenesis and Mechanism of Action of lncRNAs

lncRNAs are typical RNA molecules transcribed from exonic, intergenic, or distal eukaryotic genome regions by the RNA polymerase II, III enzymes or mitochondrial RNA polymerase IV enzyme. These molecules are processed to the mature RNA, similar to the protein-coding mRNAs. The immature RNA transcripts often undergo several processing steps for reaching the mature forms during and after transcription, such as 3’-polyadenylation, 5′-capping, splicing, and chemical base modification (24, 32).

LncRNAs can play several roles in the biological processes such as proliferation (PCNA-AS1), migration (H19), epigenetic regulation (HOTAIR), cell cycle (MALAT), cell death (MEG3), invasion (lncRNA-ATB), angiogenesis (ANRIL), autophagy (H19), apoptosis (PTENP1) and metastasis (HULC); as well as are found to be associated with several diseases such as rheumatoid arthritis, atherosclerotic coronary artery disease, diabetes, neurodegenerative disorders, and many types of cancers (32–35). As mentioned in previous section, lncRNAs, according to their mechanism of action, could classify into guide, decoy, scaffold, or signal and can modulate gene expression at the transcriptional level. As a guide, lncRNAs might activate or repress gene expression *via* relocalization of regulatory factors. Decoy lncRNAs act as an endogenous sponge for other molecules such as microRNAs and alter the bioavailability of the molecule, changing cellular function. Scaffold lncRNAs participate in the formation of ribonucleoprotein complexes. As a signal, lncRNAs could regulate gene expression (36, 37). The following are some examples of the mechanisms of action of lncRNAs.

(I) Regulating histone modifications; Important histone-modifying complexes that interact with lncRNAs include two repressive complexes: PRC1 and PRC2. X-inactive specific transcript (XIST) is a highly expressed lncRNA in females that silences gene expression by recruiting PRC2. Moreover, HOX transcript antisense RNA (HOTAIR), another lncRNA, can repress a locus named HoxD by interacting with PRC2; it can also interact with a histone demethylase and lead to developing repressive effect on chromatin by removing a histone mark. (II) Modulating DNA methylation; Tcf21 antisense RNA reducing DNA methylation (TARID) is a lncRNA involved in the mechanisms of DNA demethylation. (III) Regulating chromatin remodeling; some lncRNAs play a role in controlling chromatin remodeling complexes which can change nucleosome spacing, such as embryonic ventral forebrain-1 (evf2) and myosin heavy chain associated RNA transcripts (MHRT) (38). (IV) Interacting with transcription factors, some lncRNAs take part in signal transduction by regulating initiation, elongation, and termination of transcription factor’s actions (32). The lncRNA named rhabdomyosarcoma associated transcript (RMST) mediates the binding of the SRY-Box Transcription Factor 2 (SOX2) transcription factor to its binding sites and play a role in neuronal differentiation. (V) Looping enhancers and regulating genome organization; for instance, prostate cancer-associated noncoding RNA 1 (PRNCR1) and prostate-specific transcript (PCGEM1) are two lncRNAs that increase the enhancer-promoter looping in cancer cells (38). (VI) Regulating post-transcriptional events; includes post-transcriptional modifications such as editing, splicing, and mRNA localization (32). For example, Pnky is a neural-specific lncRNA that modulates the splicing patterns by binding to a splicing factor. (VII) Affecting development and differentiation, it has been indicated that female mice lacking XIST died during the first half of gestation (38). Another example of lncRNAs that affect development process are hematopoietic stem cell-specific lncRNAs (lncHSC). Likely lncHSC 1 and 2 participate in self-renewal, and myeloid differentiation, lncHSC6 involve in hematopoietic stem cell (HSC) differentiation, and lncHSC4 plays a role in erythroid lineage development and differentiation of blood cells. (VIII) Acting as a tumor suppressor; lncRNA growth arrest-specific 5 (Gas5) targets cancer-promoting miRNAs such as miR21 and plays a role as a tumor suppressor in T and B cell leukemia (32).

## 2 LncRNAs and Transplantation

**Figure 2:**
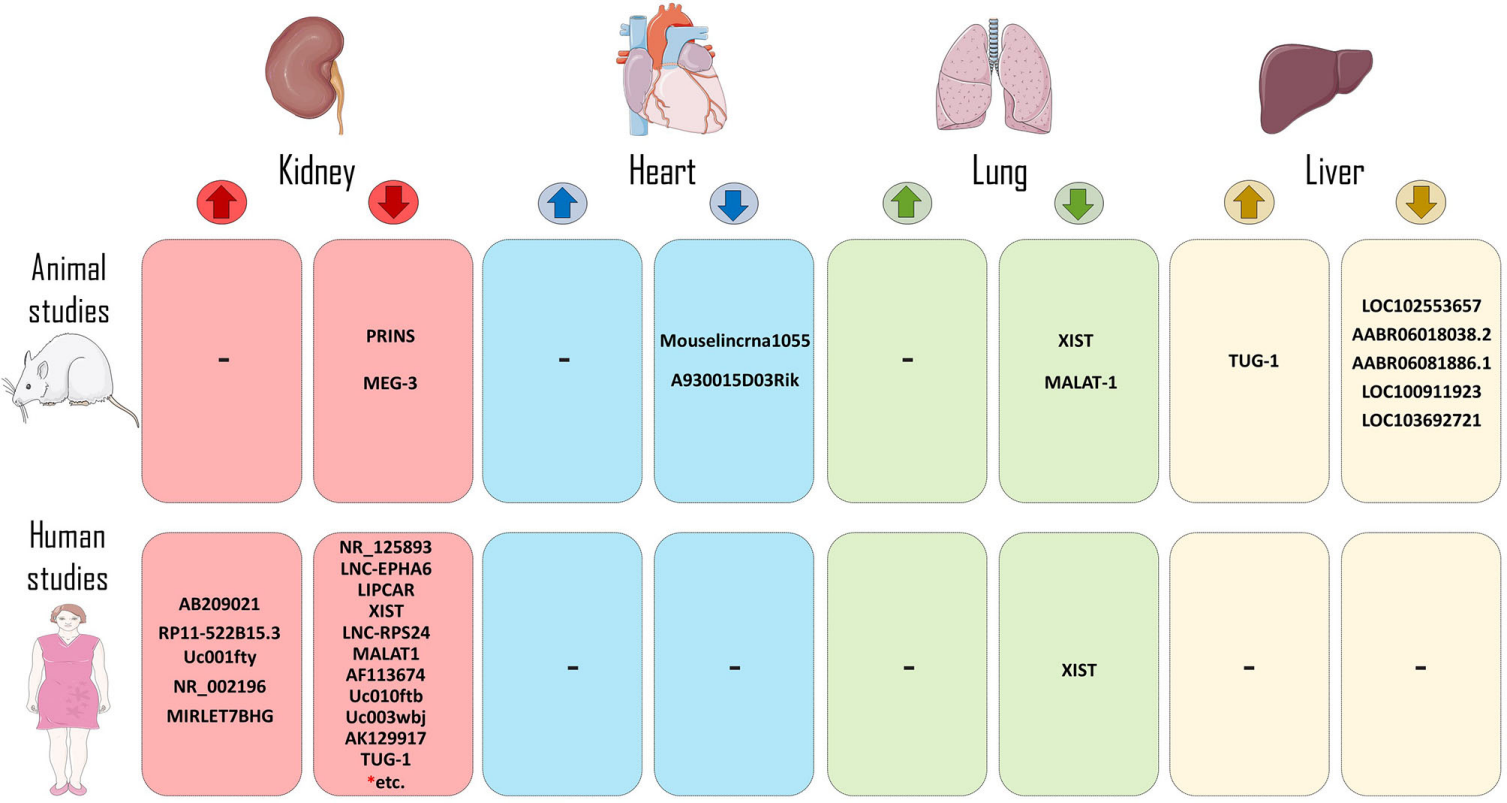
LncRNAs in the context of human and animal models of transplantation. LncRNAs that increase allograft survival (reduce rejection process) or decrease allograft survival (increase rejection incidence) have been shown in both human and animal models of transplantation. (* = Rest of LncRNAs of this group include of: RP11-354P17.15-001, RP11-395P13.3-001, AF264622, lncRNA-ATB, ENST00000628900, RP11-309P22.1, ITGB2-AS1, CARD8-AS1, MIR155HG, MIR3142HG, RP5-1171I10.5, RP11-1399P15.1, lnc-EPHA1-1.

The functions of lncRNAs in transplantation have attracted wide attentions. A growing body of literatures have investigated about the functions of several lncRNA categories in cell or organ transplantation. As the studies utilized mice/rat models or human samples, different aspects of lncRNAs activity have been assessed. Therefore, according to the design of studies, we categorized them into two categories: studies that have mainly evaluated mice/rat model of transplantation (animal studies; **Table 1**), and those which have used human samples (**Table 2** and **Figure 2**).

**Table 1:** LncRNAs in the context of animal models of transplantation.

Name of lncRNA	Chr		Sample type	Animal	Study design	Potential finding	Ref.
**Heart TX**							
Mouselincrna1055	–	–	Plasma PBLs Biopsy	Mice	Case-control	↑ in transplant acute rejection process. knockdown of these two lncRNAs: ↓ IFN-γ and TNF-α expression. ↓ IL-12Rβ1 and T-bet expression in CD4^+^ T cells. ↑ IL12Rβ1 expression in allogeneic heart GILs. ↑ in both plasma and PBLs of the allogeneic group compared to syngeneic group.	(11)
A930015D03Rik	17	Intergenic					
NEAT1	11	Intergenic	Plasma	Mice	Case-control	Alterations in the recipients of NEAT1-knockdown DCs: ↑ allograft survival. ↓infiltration of inflammatory cells in allograft. ↑ percentage of CD4^+^CD25^+^FoxP3^+^ cells in spleen. ↑ IL-10 and TGF-β in the plasma. ↓IL-12 and IL-17A in the plasma.	(39)
MALAT1	11	Intergenic		Mice		Alterations in the recipients of MALAT1 overexpressing DCs: ↑ allograft survival. ↑ number of Treg cells in spleen and cardiac allograft. ↑ serum level of IL-10. ↓Serum level of IL-6 and IL-12.	(40)
**Liver TX**							
LOC102553657			Serum Tissue	Rat	4 groups: Sham BD BDDLT Control group	↑ 7.86-fold in BDDLT compared to control group. Trans regulation of HMOX1 *via* the HNF-3β transcription factor.	(41)
AABR06018038.2						↓ 4.32-fold in BDDLT compared to control group.	
AABR06081886.1						↑ in BDDLT compared to control group. Negatively correlated with HMOX1, ATF3, TRIB3, and ANGPLT4.	
LOC100911923						↓ in BDDLT compared to control group. Positively correlated with cell apoptosis genes	
LOC103692721						Cis-regulation of IGH-6 and ABR06046430.3 involved in liver injury after BDDLT.	
TUG-1	11		Serum	Mice	Case-control	The effect of TUG-1 overexpression: ↓serum levels of ALT and AST in 3-, 7-, and 10-days post-TX. ↓ inflammatory mediators (IL-6, TNF-α, and MCP-1). ↓ the cellular apoptosis in livers.	(42)
**Lung TX**							
XIST	X	intergenic	Tissue Blood	Rat		XIST silencing → ↑ miR-21→ ↓ IL-12A→ ↑PMN apoptosis →inhibiting NET formation. ↑ miR-21 → ↓ inflammatory mediators such as IL-6, IL-8, CCL-2, and CXCL-10 → Improvement PGD after lung TX.	(43)
MALAT-1	11		Tissue Serum	Rat		MALAT-1 silencing → ↓IL-8, MPO, and ROS → ↓ Neutrophile infiltration and activation → Improvement inflammatory injury in lung TX ischemia reperfusion.	(44)
–	–	–	Tissue	Mice	8 Syngeneic TX 8 Allogeneic TX	In the allogeneic model compared to the syngeneic control group: 249 lncRNAs upregulated 212 lncRNAs downregulated 16 lncRNAs may be regulated by Myc/Max or FOXO1 transcription factor.	(45)
**Kidney TX**							
PRINS	–	–	Blood Tissue	Rat	6 iso-control 4 allo-control 4 ischemia group	↑Expression of CXCL3→↑ PRINS expression→Leading to AKI which is exacerbated graft rejection. Anti-CXCL3 → ↓PRINS → ↓ CD3^+^ T cell infiltration on day 7 post-TX→ ↓Creatinine and BUN level → Improved renal function.	(46)
MEG-3	12	Intergenic	Tissue	Mice	AllograftSham	siMEG-3 delivery to mice → MEG-3 knock-down→↑mir-181b-5p→ ↓TNF-α → ↓Tubular damage → Protection against renal injury post-TX → ↓ Serum creatinine.	(47)
**Stem cell TX**							
ENSRNOT00000052577	**-**	Intronic	Tissue	Rat	A2B5^+^ iPSC transplanted group TBI group Sham group	Up-regulated after A2B5^+^ iPSC TX → neurological function recovery	(48)
–	–	–	Tissue	Mice	Case-control	1911 downregulated and 2918 upregulated lncRNAs in hepatocytes after HSCT.	(49)
**Corneal TX**							
–	–	–	Corneal tissue	Rat	Control group Corneal autograft Corneal allograft	285 lncRNAs had differential expression in normal group and autograft group: 239 were upregulated and 46 were downregulated. 85 lncRNAs had differential expression in normal group and allograft group: 644 were upregulated and 241 were downregulated.	(50)

TX, transplantation; lncRNA, long non-coding RNA; Chr., chromosome; PBL, peripheral blood lymphocytes; GIL, graft-infiltrating lymphocyte; NEAT1, Nuclear Enriched Abundant Transcript 1; DC, dendritic cell; MALAT1, metastasis associated lung adenocarcinoma transcript 1; BD, brain dead; BDDLT, brain dead donor liver transplantation; HMOX1, Heme Oxygenase 1; HNF-3β, hepatocyte nuclear factor 3 beta; TRIB3, Tribbles homolog 3; ATF3, Activating transcription factor 3; ANGPTL4, Angiopoietin Like 4; IGH-6, immunoglobulin heavy chain 6; TUG-1, Taurine up regulated 1; ALT, alanine aminotransferase; AST, aspartate aminotransferase; HSCT, hematopoietic stem cell transplantation; TBI, traumatic brain injury; XIST, X-inactive specific transcript; PMN, polymorphonuclear; NET, neutrophil extracellular trap; PGD, primary graft dysfunction; MPO, myeloperoxidase; ROS, reactive oxygen species; BUN, blood urine nitrogen; MEG-3, maternally expressed gene 3.

**Table 2:** LncRNAs in the context of human transplantation.

Name of lncRNA	Chr*	LncRNA class*	Sample size	Sample type	Study design	Potential finding	Ref.
**Kidney TX**							
RP11-354P17.15-001 (L328)	9	Intergenic	62 aTCMR 31 SGF 10 anti- rejection therapy	Urine	Case- control	↑in aTCMR patients. Normalized with anti-rejection therapies. Associated with higher decline in GFR 1-year post-TX. IL-6 treatment of tubular epithelial cells → ↑ L327 and L328 in cell culture supernatant.	(51)
RP11-395P13.3-001 (L327)	5	Intergenic				↑in acute cellular rejection patients.	
AF264622	2	Intronic	75 pediatric (38 AR, 37 SGF) from GSE14346 75 Adult (51 AR, 19 SGF) from GSE15296	Blood	Prospective cohort (Two-year follow-up)	↑in acute rejection vs. stable patients in both pediatric and adult groups.	(10)
AB209021	3	Exon				↓in acute rejection vs. stable patients in both pediatric and adult groups.	
lncRNA-ATB	14	Exon	72 aTCMR 36 SGF	Biopsy	Case-control	↑in aTCMR patients. Inversely correlated with the renal tissue expression of miR-200c of aTCMR patients. lncRNA-ATB over-expression→ CsA-mediated apoptosis of kidney cells.	(12)
ENST00000628900 RP11-309P22.1	–	–	5 Rejection 5 SGF 5 HC	Biopsy	Case-control	↑ in rejection cases compared to SGF and HC subjects.	(11)
OIP5-AS1	15	Intergenic	22 AMR 7 TCMR 32 SGF	Blood	Case-control	↑FAS-AS1 expression level in rejection group (AMR & TCMR) in males but not females. Expression levels of other lncRNAs were not differ between two groups.	(52)
FAS-AS1	10	Antisense					
TUG1	22	Intergenic					
NEAT1	11	Intergenic					
PANDAR	6	Intergenic					
ITGB2-AS1	21	Antisense	1105 samples from: GSE36059 GSE48581 GSE21374 GSE50058	Biopsy	In silico	Two lncRNAs associate with the graft loss risk.	(14)
CARD8-AS1	19	Antisense					
MIR155HG	21	Intergenic				MIR155HG associate with AR and the graft loss risk. ↑MIR155HG expression→↑ graft rejection related pathways like GVHD, TCR and BCR signaling pathways. ↑Four lncRNAs expression level→ ↑ probability of TCMR (ORs > 1).	
MIR3142HG	5	–					
RP5-1171I10.5	17	Intergenic					
RP11-1399P15.1	2	Intergenic					
RP11-522B15.3	15	Intergenic				↑Expression level→ ↓ probability of TCMR (OR = 0.30).	
lnc-EPHA1-1 (AC093673.5)	7	Bidirectional	407 samples from: GSE57387 GSE21374 GSE25902	Biopsy	In silico	Considerably associated with the progressive chronic histological damage and graft failure → an important role in prediction of graft fibrosis and progressive fibrosis at one year after TX.	(16)
ATP1A1-AS1	1	Antisense	GSE34437, GSE75693, GSE50058, GSE76882, and GSE21374	Biopsy	In silico	The 4-lncRNA risk score model had a good sensitivity and specificity for AR of kidney allograft and prediction of the graft loss risk. The expression of these lncRNAs correlated with γT cells and eosinophils.	(53)
CTD-3080P12.3	5	Intergenic					
EMX2OS	10	Antisense					
LINC00645	14	Intergenic					
MGAT3-AS1	22	Antisense	129	PBMC	Cohort	↑ in patients with plasma Tac levels< 15µg/mL compared to those have Tac levels≥15µg/mL in first postoperative day. ↑ by 76% during the one-week post-TX. ↓in patients with DGF in comparison to patients with IGF post-TX.	(54)
MGAT3-AS1	22	Antisense	163	PBMC	Cohort	↓MGAT3-AS1/β-actin ratio →↑ Risk of BK polyomavirus (OR=3.03), and cytomegalovirus viremia in living donor kidney transplant recipients. Induction therapy with rituximab and ↓MGAT3-AS1/β-actin ratio→↑ Risk of reactivation viremia infection.	(55)
AC007114.2	17	Antisense	32 samples from: GSE86884	PBMC	In silico	There were 1063 differentially expressed lncRNAs and 3563 differentially expressed mRNAs. 466 lncRNA-mRNA pairs with cis-regulation. 5692 lncRNA-mRNA pairs with trans-regulation. These seven lncRNAs targeted ZBTB25 mRNA.	(56)
AL122035.2	14	Antisense					
AL591848.3	1	Intergenic					
IQCH-AS1	15	Antisense					
KLF3-AS1	4	Antisense					
PVT1	8	Intergenic					
SLC25A25- AS1	9	Antisense					
TMEM161B-AS1	5	Antisense				18 mRNAs such as ANGEL1, ARSK, CARF, CATSPER2, CD96, DIEXF, ELP2, HLCS were target of this lncRNA in the first week post-TX compared to pre-TX.	
–	–	–	3 AR 3 HC	Biopsy	Case- control	5339 lncRNAs have differential expression between AR and NC group: 3148 lncRNAs down-regulated. 2191 lncRNAs up-regulated. 18 lncRNAs have highest total score. 32 lncRNAs were correlated with AR pathophysiology.	(57)
AF113674	19	Intronic	3 AR 3 HC	Biopsy	In silico	Located in TSS of C3 complement→ possibly related to the innate immune response activation against allograft.	(58)
Uc010ftb	6	–				↑ in AR→ ↓ caspase 10 expression (negatively regulation caspase 10)	
Uc001fty	1	Intronic				↓ in AR Located on intronic region of CRP	
Uc003wbj	–	–				Corresponding to the TCR-β mRNA. ↑ in AR → may be related to inflammatory responses during AR process.	
AK129917	2	–				↑ in AR Located in TSS of Hsp-90	
NR_002196	–	–	25 HC 25 rejection 25 non-rejection	–	Cross section	↓ expression in the graft rejected group. ↑ in the non-rejected groups.	(59)
MIRLET7BHG (NR_110479)	22	Intergenic				↓ in the graft rejected patients. ↑ expression in non-rejected groups.	
TUG-1 (NR_002323)	22	Intergenic				↑expression level in the rejected graft patients.	
NR_125893	4	Intergenic				↑expression level in the rejected graft patients. ↓ expression levels in the non-rejected groups.	
LNC-EPHA6	3	Antisense	15 AR 32 SGF	Biopsy	Case-control	↑ Circulating level in TCMR group vs SGF patients. ↑ until six months after AR episode. ↓ one year after AR. Positively correlated with vascular injury markers sTM.	(15)
LNC-RPS24	10	Intergenic				Positively correlated with vascular injury markers sTM but not Ang-2. Similar expression level in both groups.	
LIPCAR (uc022bqs.1)	mt	Intergenic				Positively correlated with vascular injury markers sTM but not Ang-2. Similar expression level in both groups.	
XIST	X	Intergenic	18 Pre-TX 10 SGF 8 Post-TX AKI	Biopsy	In silico	Interaction with hsa-miR-212-3p and hsa-miR-122-5p → regulated the inflammation and apoptosis in AKI. As a ceRNA → regulate the expression of BRWD1 and ASF1A through sponging miR-212-3p. By sponging miR-122-5p → modulating PFKFB2 expression → may be contribute to AKI pathogenesis.	(60)
ANRIL	9	Antisense	505	Blood	Cohort	GG allele in ANRIL SNP considered a risk factor for CVE in renal transplant patients → 2.93-fold higher risk of suffering a CVE. Ischemic strokes depicted the strongest association with ANRIL rs10757278 SNP.	(13)
LIPCAR	mt	Intergenic	35 SPKT 13RTX 15HC 12 DM 14 DN	Plasma	Case- control	LIPCAR and LNC-RPS24 levels did not remarkably differ between RTX and SPKT. MALAT1 and LNC-EPHA6 → ↑ in the RTX compared with the SPKT patients. MALAT1, LIPCAR, and LNC-EPHA6→ Strongly correlated with sTM and angiogenic miRNAs→ these lncRNAs are associated with vascular injury.	(61)
LNC-RPS24	10	Intergenic					
MALAT1	11	Intergenic					
LNC-EPHA6	3	Antisense					
**Lung TX**							
XIST	X	Intergenic	42 Lung TX: 24 PGD 18 non-PGD	BALF BAL cells	Cohort	↑ in BALF and BAL cells of PGD patients ↓ miR-21 ↑ Inflammatory factors such as IL-6, IL-8, IL-12A, CCL-2, and CXCL-10 in PGC compared to non-PGD patients.	(43)
**Hematopoietic stem cell TX**							
ANRIL (CDKN2B-AS1)	9	Sense overlapping	103	Blood		rs2151280 TT genotype associated with poorer overall survival in adult patients with hematologic malignancies after allo-HSCT. rs2151280 TT → ↑ ANRIL in PBMC → ↓ P-15, P-16, and ARF → Negatively affect P53-dependent apoptosis and pRB-mediated cell cycle control.	(62)

lncRNA, long non-coding RNA; TX, transplantation; chr, chromosomal location; AR, acute rejection; HC, healthy control; aTCMR, acute T cell–mediated rejection; SGF, stable graft function; GFR, glomerular filtration rate; lncRNA-ATB, lncRNA activated by transforming growth factor β (TGF-β); AMR, antibody-mediated rejection; OIP-AS1, OIP5 antisense RNA 1; FAS-AS1, FAS Antisense RNA 1; TUG1, taurine upregulated gene 1; NEAT1, nuclear enriched abundant transcript 1; GVHD, graft versus host disease; TCR, T cell receptor; BCR, B cell receptor; OR, Odds ratio; MGAT3, β-1,4-mannosylglycoprotein 4-β-N-acetylglucosaminyltransferase antisense; Tac, tacrolimus; DGF, delayed graft function; IGF, immediate graft function; TSS, transcription start site; LIPCAR, long intergenic noncoding RNA predicting cardiac remodeling; sTM, soluble thrombomodulin; Ang-2, angiotensin 2; XIST, X-inactive specific transcript; SPKT, simultaneous pancreas kidney transplantation; RTX, renal transplantation; DM, diabetes mellitus; DN, diabetes nephropathy; AKI, acute kidney injury; ceRNA, competing endogenous RNA; BRWD1, Bromodomain And WD Repeat Domain Containing 1; ASF1A, Anti-Silencing Function 1A Histone Chaperone; PFKFB2, 6-Phosphofructo-2-Kinase/Fructose-2,6-Biphosphatase 2; ANRIL, antisense non-coding RNA in the INK4 locus; CVE, cardiovascular event; SNP, single nucleotide polymorphism; LINC01121, long intergenic non-protein coding RNA 1121; eGFR, estimated glomerular filtration rate; HTX, heart transplantation; BALF, bronchoalveolar lavage fluid; PGD, primary graft dysfunction.

^*^These items are based on information in the LNCipedia 5 database (63).

### 2.1 Animal Studies

#### 2.1.1 Heart

Some animal studies indicated that lncRNAs might be related to acute rejection episodes after cardiac transplantation. Gu et al. showed that two lncRNAs, called Mouselincrna1055 and A930015D03Rik, which were obtained in the plasma, were upregulated in mice with acute heart allograft rejection (11). lncRNAs have been linked to allograft survival *via* the functional modulation of dendritic cells (DCs) and immune tolerance induction. The evidence from previous study suggests that silencing some of the lncRNAs, such as nuclear enriched abundant transcript 1 (NEAT1), can induce tolerogenic phenotype in DCs (tol-DCs) by reducing the expression of the costimulatory molecules such as, CD80, CD86, and MHC-II through the interaction with miR-3076-3p (39). Adoptive transfer of the knocked down DCs prolonged allograft survival and has induced immune tolerance in heart transplanted mice by creating a regulatory state, including increasing the regulatory T (Treg) cells and anti-inflammatory cytokines IL-10 and TGF-β, decreasing infiltration of inflammatory cells and cytokines, and inhibiting T cell proliferation in a cardiac transplantation model (39, 64). Another lncRNA involved in the functional modulation of murine DCs is metastasis-associated lung adenocarcinoma transcript 1 (MALAT1). Wu et al. depicted that the adoptive transfusion of MALAT1-overexpressing DCs to cardiac transplanted mice can induce immune tolerance by increasing Treg cells infiltration to the cardiac allograft and enhancing splenic Tregs. Moreover, the transfer of MALAT1-overexpressing DCs elevated serum level of IL-10 and decreased IL-6 and IL-12 levels in the heart transplanted mice. Ectopic MALAT1 upregulated dendritic cell-specific intercellular adhesion molecule-3 grabbing nonintegrin (DC-SIGN) expression level through functioning as a miR155 sponge in the cytoplasm of DCs (40).

#### 2.1.2 Liver

During liver transplantation, cold-mediated hepatic damage is the main problem affecting liver graft survival rate. A high-throughput sequencing-based study has shown cold storage down-regulated taurine upregulated gene 1 (TUG1) expression, whereas TUG1 overexpression attenuates cold-mediated apoptosis *via* inhibiting intrinsic pathway of apoptosis and endoplasmic reticulum stress pathway in hepatocytes and hepatic sinusoidal endothelial cells (HSECs). Moreover, TUG-1 overexpression in mice led to massive reduction of serum levels of hepatic enzymes, alanine aminotransferase (ALT), and aspartate aminotransferase (AST) after transplantation. Therefore, overexpression of TUG1 appears to confer hepatic tissue protection and ameliorates hepatocyte functions following hepatic injury in a murine liver transplant model (42). Similar to TUG-1, AABR06081886.1 upregulation has been negatively correlated with apoptosis-related key genes such as Heme Oxygenase 1 (HMOX1), tribbles pseudo kinase 3 (TRIB3), angiopoietin like 4 (ANGPTL4), and activating transcription factor 3 (ATF3) in brain dead donor liver transplantation (BDDLT). Furthermore, it seems these genes might be involved in apoptosis triggering and liver injury after BDDLT in a rat model (41).

#### 2.1.3 Lung

lncRNAs have been shown to play a cardinal role in the pathogenesis of post lung transplantation complications including obliterative bronchiolitis (OB), primary graft dysfunction (PGD), and ischemia-reperfusion injury (IRI). For example, an integrated analysis for TFs and lncRNAs showed that eight lncRNAs such as Snhg1, Gm15290, Gm16853, Gm16761, Gm15834, AI480526, A530013C23Rik, and 5430416N02Rik had upregulated; whereas six lncRNAs including Gm10425, Gm5091, AI854517, F630040L22Rik, D130017N08Rik, and 1600020E01Rik were downregulated under the effect of Myc/Max transcription factor in mice with OB. Additionally, the expression of 9530082P21Rik and 23100001H17Rik lncRNAs were modulated *via* FOXO1 TF. Myc/Max and FOXO1 TFs might play pivotal roles in the development of OB in the murine models following lung transplantation by regulating downstream lncRNAs, mRNAs, and miRNAs (45). MALAT1 Silencing has also been demonstrated to improve the IRI in a rat model of lung transplantation by blocking neutrophils infiltration and activation through histone acetyltransferase p300-mediated downregulation of IL-8 (44). Furthermore, another recent study revealed that shRNA-mediated knockdown of XIST potentiated the apoptosis of polymorphonuclear neutrophils (PMNs) and thus prevented the formation of the neutrophil extracellular trap (NET) *via* upregulation of miR-21 and downregulation of IL-12A in a rat model of lung transplantation. Therefore, miR-21 overexpression ameliorates PGD after lung transplantation through downregulation of inflammatory mediators such as IL-6, IL-8, CCL-2, and CXCL-10 (43).

#### 2.1.4 Kidney

Acute kidney injury (AKI) induced by prolonged cold ischemia could lead to acute rejection by enhancing the recruitment of various immune cells, like T cells. Interaction of CXCR-3 and its ligand, CXCL-10, has been depicted to be involved in the T cell immigration to the allograft in a rat model of renal transplantation (65). It has been shown that CXCL-10 can upregulate the expression of chemokine-related lncRNA called psoriasis susceptibility-related RNA gene induced by stress (PRINS) in the renal allografts subjected to cold ischemia in a rat model of kidney transplantation (46). Another lncRNA that was found to be upregulated after hypoxia treatment is maternally expressed gene 3 (MEG-3). Pang et al. suggested that MEG-3 as a competing endogenous RNA (ceRNA) for miR-181b leads to upregulation of tumor necrosis factor-alpha (TNF-α) in hypoxia-mediated renal damage in the mice model of transplantation. MEG3 inhibition *via* siMEG-3 delivery promoted and reduced miR‐181b‐5p levels and TNF-α expression, respectively. Consequently, these changes protect kidney allografts from tubular damage (47). These results revealed that lncRNAs might be an important class of therapeutic targets in renal injuries after transplantation.

#### 2.1.5 Stem Cell

Lyu et al. conducted an integrated microarray analysis of lncRNAs and mRNAs in brain tissue of rats prone to traumatic brain injury (TBI). Induced pluripotent stem cells (iPSCs) -derived A2B5^+^ (A2B5^+^iPSC) cells were transplanted into the injured site of rat brain. They found 83 lncRNAs and 360 mRNAs between A2B5^+^iPSC and TBI groups differently expressed. A2B5^+^iPSC transplantation could effectively ameliorate neurological dysfunction in the brain tissue of rats in the TBI group. The mechanism of improvement is mainly related to the alteration of lncRNA and mRNA expression, specially ENSRNOT00000052577 lncRNA and its target mRNA, kinesin family member 2C (Kif2C). This study showed that ENSRNOT00000052577 and Kif2C were downregulated in the A2B5^+^iPSC group versus the TBI group (fold change 2.07 and 2.08, respectively) (48). Another study using a murine hematopoietic stem cell transplantation (HSCT) model discovered differentially expressed lncRNAs in hepatocytes using a microarray approach. The findings displayed that 1911 and 2918 lncRNAs were downregulated and upregulated, respectively, and may be involved in hepatocyte damage after HSCT. Among these dysregulated lncRNAs, there were 148/183 (down-/upregulated lncRNAs) bidirectional, 795/1473 intergenic, 209/421 intronic antisense, 304/327 natural antisense, 380/327 exon sense-overlapping, and 75/187 intronic sense-overlapping. Additionally, these lncRNAs were correlated with a dysregulation in the expression of various adjacent coding genes, such as upregulation of the T cell receptor (TCR) and downregulation of the vascular endothelial growth factor (VEGF) pathways (49).

#### 2.1.6 Cornea

The first study about the function of lncRNAs in corneal transplantation was explored by Wen et al. in the rat models of corneal allograft and autograft. They identified 162 differentially expressed lncRNAs in the allograft compared to the autograft group, among which 142 were upregulated, and 20 were downregulated, and it seems these dysregulated lncRNAs involved in the occurrence of allograft rejection 14 days after corneal transplantation (50).

### 2.2 Human Studies

#### 2.2.1 Kidney

Several LncRNAs regulate gene expression and phenotype with no alteration in the underlying DNA sequence only *via* interfering with protein activity, stability, and localization. As an important prerequisite to figure out signaling pathways in acute renal rejection, several studies have investigated the interaction between transcription factors (TFs), miRNAs, and lncRNAs by an integrated bioinformatic analysis of related datasets as the first step toward better comprehending the regulation of gene expression in acute rejection (AR) following renal transplantation (57).

The function of lncRNAs in epigenetic and gene regulation is well evidenced (66). Therefore, the finding of these studies that thousands of lncRNAs are differentially expressed in AR and healthy samples is not surprising.

Multiple lncRNAs have been shown to be dysregulated in the urine of renal transplant patients with acute T cell-mediated rejection (aTCMR). Lorenzen et al. found that three intergenic lncRNAs, RP11-354P17.15-001 (L-328), RP11-395P13.3-001 (L-327), and LNC-MYH13-3:1 (L-321), were most strongly changed in the urine of patients with acute rejection. L-328 and L-327 have upregulated in patients who experienced acute rejection compared with stable subjects. Moreover, only the expression level of L-328 decreased in patients with successful anti-rejection therapy and was also associated with a higher reduction in glomerular filtration rate (GFR) one-year after transplantation. Surprisingly, 83.61% of patients experienced subclinical rejection (i.e., no change in creatinine level) that were identifiable by L-328 expression level. Thus, in addition to aTCMR, the subclinical rejection which would have been missed by routine serum creatinine measurements could also be evaluated by L-328 level (51). In line with this study, Qiu and colleagues have also proposed that lncRNA-ATB, lncRNA activated by transforming growth factor-beta 1 (TGF-β1), could be utilized as a potential diagnostic biomarker to identify aTCMR and predict loss of renal function. This lncRNA could function as a competing endogenous RNAs (ceRNA) by competitively binding to miR-200c and thus inversely correlated with the expression level of miR-200c in renal biopsies of aTCMR patients. Moreover, treatment of human renal proximal tubule cells HK-2 with TGF-β revealed that miR-200c downregulated, while lncRNA-ATB was strongly upregulated. Meanwhile, overexpression of lncRNA-ATB induces cyclosporin A (CsA)-dependent renal cell apoptosis as well as influences on renal cell phenotype (12).

Additionally, multiple transcriptomic studies have reported a large number of dysregulated lncRNAs in whole blood (AB209021 and AF264622) (10), peripheral blood mononuclear cells (AC007114.2, AL122035.2, AL591848.3, IQCH-AS1, KLF3-AS1, PVT1, SLC25A25- AS1, and TMEM161B-AS1) (56), and tissue biopsies (ITGB2-AS1, CARD8-AS1, MIR155HG, MIR3142HG, RP5-1171I10.5, RP11-1399P15.1, RP11-522B15.3, lnc-EPHA1-1, XIST, ATP1A1-AS1, LINC00645, CTD-3080P12.3, EMX2OS, lncRNA ENST00000628900, RP11-309P22.1, AF113674, Uc010ftb, Uc001fty, Uc003wbj, and AK129917) (11,14,16,53,58,60) of renal allograft rejected patients compared to stable graft function recipients. Moreover, an acute rejection risk score model with some of these lncRNAs exhibited good diagnostic performance; for example, AB209021 and AF264622 in both pediatric and adult renal transplant patients could serve as a novel biomarker for acute rejection diagnosis (10). Zou et al. validated a risk score with a set of three related lncRNAs including ITGB2-AS1, MIR155HG, and CARD8-AS1, for predicting graft loss in transplant patients. Kidney transplant patients with higher risk scores expressed higher levels of these three lncRNAs in their biopsies (14). Also, a sensitive and specific risk score model was established using CTD-3080P12.3, ATP1A1-AS1, LINC00645, and EMX2OS that could predict allograft survival 1-, 2-, and 3-year post-transplantation (53). In a recent study, Nagarajah et al. showed that an intronic antisense lncRNA, called MGAT3-AS1, which was obtained from PBMC, was increased in patients with immediate graft function (IGF) and could thus provide information about the short-term outcome after renal transplantation. In contrast to IGF patients, the expression level of MGAT3-AS1 was downregulated in renal transplants with delayed graft function (DGF) (54). Furthermore, a lower MGAT3-AS1/β-actin ratio has been shown to increase the risk of cytomegalovirus and BK polyomavirus viremia in the first month after transplantation. Patients who had received induction therapy either with rituximab or thymoglobulin and had a lower MGAT3-AS1/β-actin depicted an elevated risk for viremia (55). A recent study showed that demographic patient parameters such as gender can affect expression levels of some lncRNAs like FAS-AS1 (52).

#### 2.2.2 Lung

XIST lncRNA was highly expressed in the bronchoalveolar lavage fluid (BALF) and BAL cells of the primary graft dysfunction (PGD) cohort. XIST acts as a regulatory sponge for miR-21 and thereby increases levels of neutrophil-infiltrating factor, such as IL-12A, a target gene for miR-21. Ultimately, by NET formation, neutrophils accelerate PGD occurrence after lung transplantation. Furthermore, reduced expression levels of miR-21 in BAL cells of PGD patients than those of non-PGD ones confirmed the ceRNA function of XIST. Additionally, the expression level of miR-21 was also negatively correlated with the grade of PGD. Moreover, the protein levels of pro-inflammatory mediators, including IL-6, IL-8, CCL-2, and CXCL10, were elevated in the PGD patients compared to non-PGD. Whilst macrophages were the main leukocyte population in the BALF of non-PGD patients, the major leukocyte population in BALF of the PGD patients were PMNs (43).

#### 2.2.3 Hematopoietic Stem Cell Transplantation (HSCT)

It has been discovered that polymorphism in ANRIL lncRNA is associated with the overall survival in adult patients with hematologic malignancies after HSCT. rs2151280 in ANRIL was related to the poorer overall survival in these patients. Conversely, none of the four investigated SNPs rs564398, rs1063192, rs2151280, and rs2157719 were associated with clinical outcomes, including the incidence of neurotoxicity, acute kidney injury (AKI), and graft versus host disease (GVHD), in these patients. The median time to death for patients who harbored rs2151280 TT was 379 days; whereas, it’s more than 1300 days for those having rs2151280 TC/CC. The underlying mechanism could be attributed to ANRIL-dependent transcription suppression on the ARF-INK4 gene. Furthermore, rs2151280 might serve as a potential prognostic biomarker for overall survival in patients suffering from hematologic malignancies after HSCT. (62).

## 3 The Potential Roles of lncRNAs as a Biomarker

Many studies have widely explored the noninvasive biomarkers for allograft rejection. For example, urinary/circulating blood concentrations of soluble adhesion molecules, chemokines, cytokines, the lymphocyte expression of granzyme B, perforin, and Fas ligand (FasL), as well as the urokinase plasminogen activator receptor (uPAR) have been assessed previously as the potential biomarkers for the allograft rejection (67). However, the clinical application of these biomarkers has not been successfully proven yet. Therefore, finding the potential biomarkers with high specificity and sensitivity, as it has been shown for some lncRNAs, can be a beneficial tool in the transplantation field. Several lncRNAs with appropriate sensitivity and specificity for detecting transplant rejection are shown in **Table 3**.

**Table 3:** Candidate lncRNA for monitoring transplantation status according to the previous literature.

Name	Group	AUC (95% CI)	Specificity	Sensitivity	*P* value	Results	Ref
RP11-354P17.15-001 (L-328)	Adult	0.76	95%	49%	< 0.001	L-328 can detect subclinical TCMR.	(51)
AF264622 AB209021	Pediatric	0.83 (0.74-0.92)	95%	66%	< 0.001	These lncRNAs exhibited an excellent performance for prediction of AR in both pediatric and adult recipients.	(10)
AF264622 AB209021	Adult	0.89 (0.82-0.96)	86%	75%			
ITGB2-AS1 MIR155HG CARD8-AS1	Adult	0.72	Nm	nm	–	3-lncRNAs were predictive for kidney graft loss.	(14)
ATP1A1-AS1 CTD-3080P12.3 EMX2OS LINC00645	Adult	0.89*	Nm	nm	–	Four lncRNAs model could predict AR and risk of renal graft loss.	(53)
MGAT3-AS1	Adult	0.83 (0.65-1.00)	86%	80%	< 0.001	Preoperative MGAT3-AS1 level could predict DGF after RTX.	(54)

AUC, area under the curve; CI, confidence interval; MGAT3, β-1,4-mannosylglycoprotein 4-β-N-acetylglucosaminyltransferase antisense; nm, not mentioned; RTX, renal transplantation, ATP1A1-AS1, ATP1A1 Antisense RNA 1; EMX2OS, EMX2 Opposite Strand; DGF, delayed graft function.

^*^1-year survival AUC = 0.89, 2-year survival AUC = 0.84, 3- year survival AUC = 0.73.

## 4 Future Landscape for the Clinical Use of lncRNAs in the Transplantation

Among various diagnostics parameters, tissue biopsy is considered a gold standard for diagnosing allograft rejection. Nevertheless, it is highly invasive and a risky procedure. Moreover, sampling errors and interobserver variations can further complicate the diagnosis of graft rejection. Therefore, identifying accurate, sensitive, and noninvasive biomarkers is essentially needed to diagnose subclinical transplant rejection. Ideally, such biomarkers could also become the cornerstone of personalized therapy. They would allow clinicians to decrease immunosuppression in patients with a low rejection risk and/or high risk of neoplastic complications or infection and also adapt the immunosuppressive protocol in high rejection risk patients. Over the past few years, many of the ncRNAs have been demonstrated to play a major function in determining the allograft status and thus have shown promise as potential therapeutic targets. Among ncRNA families, the subfamily of lncRNAs have recently emerged as the lead targets in the area of transplant research, and widespread efforts have been made towards the clinical application of these RNA-based diagnostics for monitoring the allograft loss after transplantation. It seems that these molecules can overcome the need for diagnostic biopsy and identify patients with a poor prognostic outcome of the allograft. As mentioned previously, several lncRNAs in different types of organ transplantation have been studied so far, but the transition from discovery to validation status is a major challenge, and large multi-center prospective clinical trials are now significantly required for accelerating the implementation of lncRNAs into the clinic. However, according to the recent studies (10,14,53), an interesting strategy could be using a set of lncRNAs to design a composite lncRNA-based risk score model to predict the risk of graft failure and the occurrence of complications after transplantation. These composite predictive models probably guide clinicians in choosing or adjusting the immunosuppressive drugs after transplantation.

Nonetheless, it is necessary to note that the findings described in recent studies are merely a starting point for the comprehension of functions that lncRNAs may play in transplant rejection episodes. In this context, it is worth noting that only the function of a minority of lncRNAs is been shown presently and further studies are required to characterize the exact role of lncRNAs for clinical use in transplantation.

## Author Contributions

SS: The design and conception of the study and revising the manuscript critically for important intellectual content. AS: The design and conception of the study and revising the manuscript critically for important intellectual content. SA: The design and conception of the study and revising the manuscript critically for important intellectual content. AA: The design and conception of the study and revising the manuscript critically for important intellectual content. YM: The design and conception of the study and revising the manuscript critically for important intellectual content. All authors contributed to the article and approved the submitted version.

## Conflict of Interest

The authors declare that the research was conducted in the absence of any commercial or financial relationships that could be construed as a potential conflict of interest.

## Publisher’s Note

All claims expressed in this article are solely those of the authors and do not necessarily represent those of their affiliated organizations, or those of the publisher, the editors and the reviewers. Any product that may be evaluated in this article, or claim that may be made by its manufacturer, is not guaranteed or endorsed by the publisher.
